# Association between tonsillitis and newly diagnosed ankylosing spondylitis: A nationwide, population-based, case-control study

**DOI:** 10.1371/journal.pone.0220721

**Published:** 2019-08-01

**Authors:** Wen-Cheng Chao, Ching-Heng Lin, Yi-Ming Chen, Rong-San Jiang, Hsin-Hua Chen

**Affiliations:** 1 Department of Medical Research, Taichung Veterans General Hospital, Taichung, Taiwan; 2 Division of Chest Medicine, Department of Internal Medicine, Taichung Veterans General Hospital, Taichung, Taiwan; 3 Department of Nursing, College of Medicine & Nursing, Hung Kuang University, Taichung, Taiwan; 4 Department of Business Administration, National Changhua University of Education, Changhua, Taiwan; 5 Department of Healthcare Management, National Taipei University of Nursing and Health Sciences, Taipei, Taiwan; 6 Department of Public Health, College of Medicine, Fu Jen Catholic University, New Taipei City, Taiwan; 7 Division of Allergy, Immunology and Rheumatology, Department of Internal Medicine, Taichung Veterans General Hospital, Taichung, Taiwan; 8 Institute of Biomedical Science and Rong Hsing Research Center for Translational Medicine, Chung-Hsing University, Taichung, Taiwan; 9 School of Medicine, National Yang-Ming University, Taipei, Taiwan; 10 Department of Otolaryngology, Taichung Veterans General Hospital, Taichung, Taiwan; 11 School of Medicine, Chung-Shan Medical University, Taichung, Taiwan; 12 Institute of Public Health and Community Medicine Research Center, National Yang-Ming University, Taipei, Taiwan; 13 Department of Industrial Engineering and Enterprise Information, Tunghai University, Taichung, Taiwan; Karolinska Institutet, SWEDEN

## Abstract

**Objectives:**

To investigate the association between tonsillitis and the risk of newly diagnosed ankylosing spondylitis (AS).

**Methods:**

We used 2003–2012 data from Taiwan’s National Health Insurance Research Database to conduct this nationwide, population-based, case-control study. We identified AS patients newly diagnosed between 2005 to 2012 as the study group and selected age, sex and index-year matched (1:6) non-AS individuals as controls. The association between tonsillitis and risk of newly diagnosed AS was determined by calculating odds ratios (ORs) with 95% confidence intervals (CIs) using conditional logistic regression analysis.

**Results:**

We identified 37,002 newly diagnosed AS cases and 222,012 matched non-AS controls. Patients with AS were more likely to have tonsillitis (aOR 1.46, 95% CI 1.43–1.50), appendicitis (aOR 1.29, 95% CI 1.13–1.48) and periodontitis (aOR 1.35, 95% CI 1.31–1.38) than non-AS control subjects. The association between tonsillitis and AS was consistent using varying definitions for tonsillitis, and we further found that a high frequency of visits for tonsillitis, a high medical cost for tonsillitis and a long interval between diagnosis were associated with newly diagnosed AS in a dose-response manner. Furthermore, the association between tonsillitis and AS appeared to be stronger in females (aOR 1.59, 95% CI 1.53–1.65) than those in males (aOR 1.39, 95% CI 1.35–1.44).

**Conclusions:**

The present study revealed an association between AS risk and prior tonsillitis and indicates the need for vigilance of AS-associated symptoms in patients who had been diagnosed with tonsillitis, particularly in females.

## Introduction

Ankylosing spondylitis (AS), an autoimmune arthritis, is characterized by a dysregulated inflammation involving mainly the sacroiliac joints, axial structure, and enthesis [1]. AS develops before the age of 40 and the global prevalence of AS ranges from 7.4 to 31.9 per 10,000 people [2]. The exact mechanisms underlying AS remains unknown, and genetic predisposition including HLA-B27, gut dysbiosis, and environmental triggers including acute infectious diseases have been implicated in the pathogenesis of AS [3–5]. Tonsillitis is a leading disease in otolaryngology, and the admission for tonsillitis was found to rise by 310% between 2003 and 2011 in the United Kingdom [6, 7]. Notably, tonsillitis has recently been implicated in the development of autoimmune arthritis including reactive arthritis and rheumatoid arthritis (RA), and gut dysbiosis appears to play an essential role in the linkage between infectious diseases and autoimmune arthritis [8–10]. However, the data regarding AS and tonsillitis, particularly in the adulthood tonsillitis, remains limited [11]. We thus aimed to address the correlation between infectious diseases including tonsillitis and the newly diagnosed AS using a nationwide, population-based claim database.

## Materials and methods

### Ethical statements

This study was approved by the Institutional Review Board of Taichung Veterans General Hospital, Taiwan (IRB number: CE16251A-1). All the personal data obtained were anonymized before analysis, and informed consent was thus waived.

### Data source

In Taiwan, a single-payer National Health Insurance (NHI) program was launched on March 1, 1995. As of 2015, up to 99.6% of Taiwan’s population was enrolled in the NHI program [12]. We used the National Health Insurance Research Database (NHIRD), which contains medical claims, including inpatient and outpatient medical records of all enrollees. The National Health Research Institutes is responsible for the management of the NHIRD and releases the data for research purpose. Moreover, the NHIRD has constructed a representative database of 1,000,000 individuals, known as the Longitudinal Health Insurance Database (LHID2000), which comprises patients randomly selected from all enrollees who received services in 2000.

### Study samples

#### Newly diagnosed AS patients identified from the entire Taiwanese population

In this study, AS patients were defined as having at least three ambulatory visits or one hospital admission with a diagnosis of AS (International Classification of Diseases, 9^th^ Revision, Clinical Modification (ICD-9-CM) code 720 or 720.00) during 2003–2012, and those who had AS diagnosis before 2005 were excluded given that we aimed to identify those with newly diagnosed AS. To ensure diagnostic validity, we only included those who had received medications, including corticosteroid, non-steroidal anti-inflammatory drugs, sulfasalazine, methotrexate, and biological agents including adalimumab, etanercept, and golimumab. The first date of a visit with an AS diagnosis was selected as the index date, and the index year was the year of the index date.

#### Matched non-AS individuals selected from representative sample of one million

From the LHID2000, we randomly selected non-AS individuals, matching AS cases (1:6) for age, gender, and the index year after exclusion of individuals who ever had ICD-9 codes for AS. The index date used for non-AS controls was the day of the first ambulatory visit for any reason in the index year.

### Definitions of tonsillitis-associated diagnoses

We used both inpatient and outpatient databases in this study and defined tonsillitis by one ambulatory visit or hospital admission with the tonsillitis-associated diagnosis. The codes of tonsillitis-associated diagnoses and procedures included acute tonsillitis (ICD-9-CM code 463), chronic tonsillitis (ICD-9-CM code 474.0), and tonsillectomy (procedure code 28.0–28.3, 28.7–28.9).

### Potential confounders

Potential confounders used for adjustment in the conditional logistic regression included age, gender, Charlson comorbidity index (CCI), history of periodontitis, and history of appendicitis. The presence of comorbidity was defined as having at least three ambulatory visits or one inpatient visit with a corresponding ICD-9CM code within 1 year before the index date. The CCI, as adapted by Deyo *et al*. [13]. was utilized to assess the level of general comorbid medical conditions. Additionally, we also included periodontitis and appendicitis (ICD-9-CM code 540–543 or procedure code for appendectomy 47.0) as covariates given that previous studies, including our investigations, found that periodontitis and appendicitis were associated with autoimmune arthritis [14–16]. To minimize the potential underestimation of remote periodontitis and appendicitis, we applied the same definition used in tonsillitis and included the diagnosis of periodontitis and appendicitis during 2003–2012. Periodontitis was defined by the ICD9-CM codes (523.3–5) and concurrently received antibiotic therapy, or periodontal treatment other than scaling, or scaling more than twice per year by certified dentists [14]. In the present study, we only recorded exposures, including tonsillitis, appendicitis and periodontitis, prior to the first AS-diagnosis to ensure the causal effect.

### Statistical analysis

Data were presented as the mean ± standard deviation (SD) for continuous variables and as number (percentages) for categorical variables. The differences were analyzed using Student’s *t*-test for continuous variables and Pearson’s χ^2^ test for categorical variables. A conditional logistical regression model was conducted to estimate the odds ratio (OR) and 95% confidence interval (CI) of newly diagnosed AS after adjustment for age, gender, CCI, history of periodontitis, and history of appendicitis. All the data were analyzed using SAS statistical software version 9.3 (SAS Institute, Inc., Cary, NC, USA). A P value <0.05 was considered statistically significant.

## Results

### Characteristics of the study population

A total of 37,002 newly diagnosed AS patients were identified and 222,012 matched non-AS control subjects were assessed (see S1 Dataset for details). We found that AS case subjects had a higher CCI (0.37±0.91 vs. 0.23±0.74, p<0.001) and were more likely to have tonsillitis (45.5% vs. 36.2%, p<0.001), appendicitis (0.7% vs. 0.5%, p<0.001), and periodontitis (29.0% vs. 22.8%, p<0.001) (Table 1). The interval between the diagnosis of infectious diseases, including appendicitis, periodontitis and tonsillitis, and the development of AS were 3.4±2.3, 3.2±2.2, 3.8±2.4 years, respectively.

**Table 1:** Demographic data and clinical characteristics among patients.

	Control		Case	
	(*n* = 222,012)		(*n* = 37,002)	P-value
**Age, years**	42.6 ± 17.1		42.6 ± 17.1	>0.999
**Age group**				>0.999
≤45 years	130,680 (58.9)		21,780 (58.9)	
> 45 years	91,332 (41.1)		15,222 (41.1)	
**Gender**				>0.999
Female	81,522 (36.7)		13,587 (36.7)	
Male	140,490 (63.3)		23,415 (63.3)	
**CCI**	0.23 ± 0.74		0.37 ± 0.91	<0.001
**CCI group**				<0.001
0	192,289 (86.6)		28,573 (77.2)	
≥1	29,723 (13.4)		8,429 (22.8)	
**Appendicitis**	1,187 (0.5)		269 (0.7)	<0.001
Interval between appendicitis and AS	NA		3.4 ± 2.3	
**Periodontitis**	50,614 (22.8)		10,716 (29.0)	<0.001
Interval between periodontitis and AS	NA		3.2 ± 2.2	
**Tonsillitis**	80,316 (36.2)		16,852 (45.5)	<0.001
Interval between tonsillitis and AS	NA		3.8 ± 2.4	
** Number of visits for tonsillitis**				<0.001
No tonsillitis	141,696 (63.8)		20,150 (54.5)	
1 visit	31,799 (14.3)		5,772 (15.6)	
2 visits	15,569 (7.0)		3,165 (8.6)	
3–4 visits	15,252 (6.9)		3,288 (8.9)	
>4 visits	17,696 (8.0)		4,627 (12.5)	
** Cumulative cost of tonsillitis-related visits (New Taiwan dollars)**				<0.001
No tonsillitis	141,696 (63.8)		20,150 (54.5)	
Q1 (0< dollars ≤396)	21,312 (9.6)		3,829 (10.3)	
Q2 (396< dollars ≤770)	19,754 (8.9)		3,760 (10.2)	
Q3 (770< dollars ≤1,611)	19,929 (9.0)		4,293 (11.6)	
Q4 (>1,611 dollars)	19,321 (8.7)		4,970 (13.4)	
** Interval between the tonsillitis and AS**				<0.001
No tonsillitis	141,696 (63.8)		20,150 (54.5)	
Q1 (0≤ years <1.8)	21,341 (9.6)		3,981 (10.8)	
Q2 (1.8≤ years <3.3)	19,722 (8.9)		3,987 (10.8)	
Q3 (3.3≤ years <5.4)	20,154 (9.1)		4,300 (11.6)	
Q4 (≥5.4 years)	19,099 (8.6)		4,584 (12.4)	

Abbreviation: CCI, Charlson comorbidity index; AS, ankylosing spondylitis; NA, not available.

### Association between tonsillitis, periodontitis as well as appendicitis and the risk of ankylosing spondylitis

We investigated the association between the risk of AS and infectious diseases including tonsillitis, appendicitis, and periodontitis. A conditional logistic regression model that was adjusted for potential confounders revealed that AS case subjects were more likely to have tonsillitis (aOR 1.46, 95% CI 1.43–1.50), appendicitis (aOR 1.29, 95% CI 1.13–1.48) and periodontitis (aOR 1.35, 95% CI 1.31–1.38) than non-AS control subjects (Table 2). Next, we conducted a sensitivity analysis by stratifying the diagnosis of tonsillitis with acute and chronic tonsillitis, and found a similar strength of correlation between newly diagnosed AS and acute tonsillitis (aOR 1.46, 95% CI 1.43–1.50) as well as chronic tonsillitis (aOR 1.52, 95% CI 1.36–1.70) (Table 3). Taken together, these findings showed a positive correlation between newly diagnosed AS and infectious diseases, including tonsillitis, appendicitis as well as periodontitis and found a consistent risk for AS in those with acute and chronic tonsillitis.

**Table 2:** Unadjusted and adjusted odds ratios of association between variables and risk of ankylosing spondylitis.

	Univariate	Multivariate
	OR (95% CI)	aOR (95% CI)
**CCI≥1**	2.22 (2.15–2.29)	2.14 (2.08–2.21)
**Periodontitis**	1.42 (1.38–1.45)	1.35 (1.31–1.38)
**Appendicitis**	1.36 (1.19–1.56)	1.29 (1.13–1.48)
**Tonsillitis**	1.52 (1.49–1.56)	1.46 (1.43–1.50)

Matched variables include age, sex and year of index date. Abbreviation: OR, odds ratio; CI, confidence interval; CCI, Charlson comorbidity index.

**Table 3:** Unadjusted and adjusted odds ratios of association between variables and risk of ankylosing spondylitis.

	Univariate	Multivariate
	OR (95% CI)	aOR (95% CI)
**Tonsillitis** (ICD9:474.0, 463)	1.52 (1.49–1.56)	1.46 (1.43–1.50)
Acute tonsillitis (ICD9:463)	1.52 (1.49–1.56)	1.46 (1.43–1.50)
Chronic tonsillitis (ICD9:474.0)	1.63 (1.45–1.82)	1.52 (1.36–1.70)
**Number of visits for tonsillitis**		
No tonsillitis	1.00 (reference)	1.00 (reference)
1 visit	1.31 (1.27–1.35)	1.28 (1.24–1.32)
2 visits	1.48 (1.42–1.54)	1.43 (1.37–1.49)
3–4 visits	1.59 (1.52–1.65)	1.52 (1.46–1.59)
>4 visits	1.96 (1.89–2.03)	1.82 (1.75–1.89)
**Cumulative cost of tonsillitis-related visits (New Taiwan dollars)**		
No tonsillitis	1.00 (reference)	1.00 (reference)
Q1 (0< dollars ≤396)	1.30 (1.25–1.35)	1.27 (1.22–1.32)
Q2 (396< dollars ≤770)	1.38 (1.33–1.44)	1.34 (1.29–1.39)
Q3 (770< dollars ≤1,611)	1.58 (1.52–1.64)	1.52 (1.47–1.58)
Q4 (> 1,611 dollars)	1.92 (1.85–1.99)	1.79 (1.72–1.85)
**Interval between the first tonsillitis visit and the index date**		
No tonsillitis	1.00 (reference)	1.00 (reference)
Q1 (0≤ years <1.8)	1.31 (1.26–1.36)	1.27 (1.22–1.32)
Q2 (1.8≤ years <3.3)	1.44 (1.38–1.49)	1.38 (1.33–1.44)
Q3 (3.3≤ years <5.4)	1.60 (1.54–1.67)	1.53 (1.48–1.59)
Q4 (≥5.4 years)	1.93 (1.85–2.01)	1.84 (1.76–1.91)

Adjusted variables include periodontal disease, CCI, and appendicitis. Abbreviation: OR, odds ratio; CI, confidence interval; CCI, Charlson comorbidity index

### Impacts of tonsillitis severity and the interval between tonsillitis and ankylosing spondylitis

We then stratified the frequency of visit for tonsillitis and medical cost of tonsillitis to explore whether the strength of correlation between tonsillitis and AS was associated with the severity of tonsillitis. AS risk was strongest in those with the highest number of visits for tonsillitis (>4 visits) (aOR 1.82, 95% CI 1.75–1.89) followed by risks in those with 3–4 visits (aOR 1.52, 95% CI 1.46–1.59), those with 2 visits (aOR 1.43, 95% CI 1.37–1.49), and those with 1 visit (aOR 1.28, 95% CI 1.24–1.32). With regard to the medical cost, a similar pattern was found between newly diagnosed AS and the four quartiles categorized by medical cost for tonsillitis. The strongest risk for AS was observed in those with the highest number of medical cost for tonsillitis (Q4) (aOR 1.79, 95% CI 1.72–1.85) followed by risks in Q3 (aOR 1.52, 95% CI 1.47–1.58), Q2 (aOR 1.34, 95% CI 1.29–1.39), and Q1 (aOR 1.27, 95% CI 1.22–1.32). Furthermore, we stratified the interval (years) between diagnosis-date of tonsillitis and AS into four quartiles and identified that a longer interval was associated with a stronger association between tonsillitis and newly diagnosed AS. In detail, AS risk was the strongest among those with the longest interval between tonsillitis and AS (Q4) (aOR 1.84, 95% CI 1.76–1.91) followed by risks in Q3 (aOR 1.53, 95% CI 1.48–1.59), Q2 (aOR 1.38, 95% CI 1.33–1.44), and Q1 (aOR 1.27, 95% CI 1.22–1.32) (Table 3). Additionally, to examine the potential effect of interaction among variables, we further divided the subjects by age (< and ≥ 45 years) gender, CCI (0 and ≥ 1), periodontitis, and appendicitis (Table 4). We noted a slightly stronger risk for AS (aOR 1.59, 95% CI 1.53–1.65) in female patients with tonsillitis compared with those in male tonsillitis patients (aOR 1.39, 95% CI 1.35–1.44) (see S1 Table for details). Collectively, these data demonstrate a consistent correlation between tonsillitis and newly diagnosed AS, particularly in females.

**Table 4:** Analysis of the interaction effects among variables on the correlation between tonsillitis and risk of newly diagnosed ankylosing spondylitis.

	P for interaction
**Tonsillitis** x **Age** (≤45 vs. > 45 years)	0.177
**Tonsillitis** x **Gender**	<0.001
**Tonsillitis** x **CCI group** (0 vs. ≥1)	0.023
**Tonsillitis** x **Periodontitis**	0.653
**Tonsillitis** x **Appendicitis**	0.227

Abbreviation: CCI, Charlson comorbidity index

## Discussion

In this population-based study, we investigated the correlation between tonsillitis and newly diagnosed AS. We found that tonsillitis, appendicitis and periodontitis were independently associated with newly diagnosed AS. Additionally, we demonstrated that the severity of tonsillitis and interval between diagnosis of tonsillitis and AS correlated with the strength of association between tonsillitis and newly diagnosed AS. Furthermore, the association between tonsillitis and AS appeared to be stronger in females than in males. These findings highlight the correlation between tonsillitis and AS and indicate the need for vigilance of AS-associated symptoms in patients who had been diagnosed with tonsillitis, particularly in females.

The underlying mechanisms of development or exacerbation of autoimmunity in AS are complex, involving genetic predisposition including HLA-B27, gut dysbiosis, and environmental triggers including infectious diseases. The tonsils are secondary lymphoid tissue in humans, and tonsillitis, including acute tonsillitis and chronic tonsillitis, is a leading disease in otolaryngology [6]. Indeed, it remains controversial as to whether tonsils contribute to infection control or merely represent futile immune entities [17, 18]. Palomares *et al*. recently demonstrated that tonsils were involved in the tolerance to allergens through the generation of allergen-specific FOXP3^+^ regulatory T cells, indicating that tonsils are crucial in the development of immune-tolerance [19]. We thus postulate that the alteration of immune tolerance in patients with tonsillitis might lead to dysregulated inflammation in autoimmune arthritis including AS; therefore, tonsillitis might lead to the diagnosis of AS due to the exacerbated spondylitis.

Increasing evidence suggests that gut microbiota plays a pathogenic role in autoimmune arthritis including RA and AS [10]. Mouse studies have found that the gut microbiota is involved in the development of autoimmune arthritis as evidenced by the reduced severity and incidence of autoimmune arthritis in germ-free mice [20]. Furthermore, Ciccia *et al*. demonstrated a correlation between alteration of gut microbiota, i.e., gut dysbiosis, and systemic inflammation through IL-22/23 signaling in patients with AS [21, 22]. Moreover, the increased intestinal permeability, probably predisposed by genetic susceptibility including HLA-B27 expression, was found to elicit continuous antigenic stimulation with activation of effector T cells in patients with AS [4, 23]. Notably, one recently published study demonstrated that microbial infection at mucosal sites may elicit a break in immune tolerance by promoting the presentation of self-antigens derived from epithelial cell apoptotic bodies, and this crucial finding indicates that microbial infection may be involved in the initiation of autoimmunity [24]. Taken together, the aforementioned evidence highlights the key role of the gut microbiota in autoimmune arthritis, and tonsillitis may be associated with gut dysbiosis through a number of pathways, including the overgrowth of pathogenic microbial of tonsillitis, the use of antibiotics for tonsillitis, and the alteration of immunological responses [19]. Therefore, we postulated that tonsillitis might be associated with the dysregulated inflammation in spondylitis through gut dysbiosis.

In the present study, we also found that periodontitis and appendicitis were independent risk factors for AS. The positive correlation between AS and periodontitis as we shown in this study was consistent with the results of a previous study which included 6,821 patients with AS obtained from a database of 1,000,000 randomly selected individuals in Taiwan (LHID200 of NHIRD) [15], while the present study included all of the newly diagnosed patients with AS between 2003 and 2012 in Taiwan (n = 37,002). With regard to appendicitis, a divergent association between appendicitis and immunological diseases has been reported [25, 26]. In inflammatory bowel diseases, appendectomy was found to be positively associated with Crohn’s disease but inversely associated with ulcerative colitis [25, 26], and the complex factors including age and alteration of the gut microbiota may account for the distinct risk of inflammatory bowel diseases among patients who received appendectomy [27]. In line with our study in AS, one Taiwanese study found an increased risk of RA (HR 1.61, 95% CI 1.05–2.48) among adult patients underwent appendectomy [16]. Additionally, given the relatively long interval between the diagnosis of appendicitis as well as periodontitis and the development of AS (Table 1), we think that appendicitis and periodontitis should be more likely the independent risk for AS, instead of merely triggering the development of AS in patients who had tonsillitis. Intriguingly, Lindström *et al*. conducted a nationwide case-control study in Sweden and found a decreased risk of AS in patients underwent an appendectomy in the childhood [11]. Children and adults appear to have distinctly different gut microbiota [28, 29], which might explain at least in part the discordant risk for AS between childhood and adulthood appendicitis. In addition to age, gender has also been implicated as a factor involved in the reciprocal regulatory immune functions, sexual hormones and microbiota composition in autoimmune diseases [30, 31], and our previous study had found that men tend to have a higher cumulative AS-associated healthcare utilization than women [32]. In the present study, we noted a stronger correlation between tonsillitis and newly diagnosed AS in females compared with that in males, and we speculate that this gender-associated distinct risk for AS in those with tonsillitis might result from gender-associated gut dysbiosis.

Lindström *et al*. conducted a nationwide case-control study in Sweden and reported that childhood hospitalization for tonsillitis was associated with the development of AS in the adulthood (OR 1.31, 95% CI 1.03–1.67) [11]. To specifically address the impact of remote tonsillitis on newly diagnosed AS, Lindström *et al*. included patients with tonsillitis that developed before the age of 17 years in their research and suggested that tonsillitis may be implicated in the development of AS. Given that the alteration of immune balance resulting from tonsillitis may lead to either the initiation of autoimmunity or dysregulated inflammation in individuals with existed spondylitis, we thought that both remote and recent tonsillitis should be taken into account to address the association between tonsillitis and AS. In the present study, we enrolled patients with tonsillitis prior to the diagnosis of AS and both recent and remote tonsillitis were hence included in our study. Additionally, Lindström *et al*. used an inpatient database in their study, while in the present study we used both inpatient and outpatient data which are available in the NHIRD. Therefore, and it is thus reasonable that the strength of correlation in our study (OR 1.46, 95% CI 1.43–1.50) was slightly higher than that reported in the study by Lindström *et al*.

This study has limitations. First, owing to the nature of the study design, it was not possible to demonstrate a causal link between tonsillitis and newly diagnosed AS; however, increasing evidence support the essential role of the gut microbiome in the linkage between infectious diseases and autoimmunity [4, 10]. Second, the clinical severity of tonsillitis was not assessed in this study; however, we used frequency of visits for tonsillitis and medical cost as a proxy of tonsillitis severity and demonstrated a dose-response effect between tonsillitis and AS. Third, the diagnosis of AS could not be validated although the prevalence of AS in the present study was similar to that reported in previous studies conducted in Taiwan using the NHIRD [15, 33].

In conclusion, we identified an increased risk for AS in patients with tonsillitis using a population-based claim database. There was a stronger correlation between tonsillitis and AS in patients with a higher severity of tonsillitis or a longer interval between diagnosis of tonsillitis and AS. These findings indicate that tonsillitis may trigger a gradual exacerbation of inflammation among patients with AS. More studies are warranted to explore the underlying mechanisms linking tonsillitis with AS and to investigate the roles of tonsillitis in other autoimmune diseases.

## Supporting information

S1 Dataset (CSV)

S1 Table Details of the interaction effects among variables on the correlation between tonsillitis and risk of newly diagnosed ankylosing spondylitis. (DOCX)
