# Supplementary material for: Association between tonsillitis and newly diagnosed ankylosing spondylitis: A nationwide, population-based, case-control study
Source: PLoS One. 2019 Aug 1;14(8):e0220721. doi: 10.1371/journal.pone.0220721 (PMC6675079; doi:10.1371/journal.pone.0220721)
Supplement: S1 Table — (DOCX) [file pone.0220721.s002.docx]

**S1 Table. Details of the interaction effects among variables on the correlation between tonsillitis and risk of** **newly diagnosed ankylosing spondylitis**

|  | OR (95%CI) | P value | P for interaction |
| --- | --- | --- | --- |
| **Age group** |  |  | 0.177 |
| ≤45 years | 1.43 (1.39–1.48) | <0.001 |  |
| > 45 years | 1.51 (1.45–1.56) | <0.001 |  |
| **Gender** |  |  | <0.001 |
| Female | 1.59 (1.53–1.65) | <0.001 |  |
| Male | 1.39 (1.35–1.44) | <0.001 |  |
| **CCI group** |  |  | 0.023 |
| 0 | 1.46 (1.42–1.50) | <0.001 |  |
| ≥1 | 1.47 (1.37–1.58) | <0.001 |  |
| **Periodontal disease** |  |  | 0.653 |
| No | 1.47 (1.43–1.52) | <0.001 |  |
| Yes | 1.39 (1.32–1.47) | <0.001 |  |
| **Appendicitis** |  |  | 0.227 |
| No | 1.47 (1.43–1.50) | <0.001 |  |
| Yes | 0.45 (0.03–8.20) | 0.591 |  |

Abbreviation: CCI, Charlson comorbidity index; OR, odds ratio; CI, confidence interval.
